# The role of the small GTPase Rab31 in cancer

**DOI:** 10.1111/jcmm.12403

**Published:** 2014-12-03

**Authors:** Christelle En Lin Chua, Bor Luen Tang

**Affiliations:** a Department of Biochemistry, Yong Loo Lin School of Medicine, National University of SingaporeSingapore; b NUS Graduate School for Integrative Sciences and Engineering, National University of SingaporeSingapore

**Keywords:** cancer, mucin1, Rab31, membrane traffic

## Abstract

Members of the small GTPase family Rab are emerging as potentially important factors in cancer development and progression. A good number of Rabs have been implicated or associated with various human cancers, and much recent excitement has been associated with the roles of the Rab11 subfamily member Rab25 and its effector, the Rab coupling protein (RCP), in tumourigenesis and metastasis. In this review, we focus on a Rab5 subfamily member, Rab31, and its implicated role in cancer. Well recognized as a breast cancer marker with good prognostic value, recent findings have provided some insights as to the mechanism underlying Rab31's influence on oncogenesis. Levels of Oestrogen Receptor α (ERα)- responsive Rab31 could be elevated through stabilization of its transcript by the RNA binding protein HuR, or though activation by the oncoprotein mucin1-C (MUC1-C), which forms a transcriptional complex with ERα. Elevated Rab31 stabilizes MUC1-C levels in an auto-inductive loop that could lead to aberrant signalling and gene expression associated with cancer progression. Rab31 and its guanine nucleotide exchange factor GAPex-5 have, however, also been shown to enhance early endosome-late endosome transport and degradation of the epidermal growth factor receptor (EGFR). The multifaceted action and influences of Rab31 in cancer is discussed in the light of its new interacting partners and pathways.

- Introduction
- Rabs in human cancer
- Rab31 and findings implicating Rab31 in human cancers
- Mechanisms underlying Rab31's role in cancer
  – Why are Rab31 levels elevated in cancer cells?
  – Is Rab31 a driver in cancer, and if so, how?
  – Rab31's modulation of EGFR trafficking – could it be tumor suppressive?
- Epilogue and future perspectives

## Introduction

There is an enormous flux of both membrane and soluble cargoes between intracellular membranous compartments in the eukaryotic cell. It is necessary for this flow of multidirectional traffic to be adequately regulated, and dysregulation at any point of the trafficking network could lead to disease states. A particularly important group of proteins regulating membrane traffic in eukaryotic cells are the Sar/Arf family and the Rab family of small GTPases [1]. Rabs (Ras-related proteins in brain) [2,3] constitute the largest subfamily of the Ras superfamily of small GTPases [4], and has more than 60 genes encoded within the human genome. Translated as soluble, cytosolic proteins, Rabs acquire C-terminal prenylated lipid anchors (geranylgeranylation) and are localized rather specifically to different sub-cellular compartments for the regulation of particular membrane trafficking step(s) in the exocytic and endocytic pathways [5–7].

Analogous to the function of the proto-oncogene Ras in cellular signalling [8], Rab proteins are post-translationally modified and go through a cycle of guanine nucleotide exchange and hydrolysis to act as key switches in membrane traffic pathways [2,9]. Rabs are kept in the cytosol by GDP dissociation inhibitors (GDIs) [10]. The Rab escort protein engages cytosolic Rab proteins to be presented to Rab geranylgeranyl transferase [11], allowing the Rab protein to be geranylgeranylated at its C-terminal cysteine residues before it is escorted to the target membrane [12,13]. The added lipid anchor facilitates the attachment of a Rab protein to the target membrane, but full activation requires its bound GDP to be exchanged for GTP, a process that is facilitated by guanine nucleotide exchange factors (GEFs) [14,15]. Activated, GTP-bound Rabs engage effector molecules such as tethering complexes [16], motor proteins and motor adaptors [17], as well as components of the vesicle fusion machinery [18] to facilitate tethering and docking of vesicles to their target membranes. Rabs have also been shown to interact directly or indirectly with some cargo molecules [19]. Rab proteins are inactivated when the bound GTP is hydrolysed back to GDP. This will not occur spontaneously to any significant degree as Rabs have intrinsically weak GTPase activities. Instead, inactivation by GTP hydrolysis is assisted or regulated by GTPase activating proteins (GAPs) [14,20].

Given the critical importance of regulating membrane traffic which impinges on tightly regulated processes like cell proliferation and cell migration, it is expected that Rab small GTPases' activities gone awry may directly or indirectly contribute to human diseases. Several Rab genes are associated to heritable monogenic diseases. Rab7 mutations underlie Charcot–Marie–Tooth type 2B neuropathy, a peripheral nervous system disorder that is believed to be due, in part, to the dysregulation of peripherin (a neuronal intermediate filament that has been shown to interact with Rab7) [21,22]. Rab18 mutations [23] and that of a putative RabGAP TBC1D20 [24] causes Warburg micro-syndrome, a rare autosomal recessive genetic disorder characterized by microcephaly, defects in the visual system and mental retardation. Mutations in Rab23, which plays a role in the regulation of Sonic Hedgehog (Shh) signalling, underlie another autosomal recessive disorder, Carpenter's syndrome, characterized by craniosynostosis, polysyndactyly, obesity and cardiac defects [25,26]. Rab27, which plays a role in melanosome transport *via* its effector myosin Va, has been implicated in Griscelli syndrome type 2, a recessive disorder in which patients exhibit pigmentation defects like partial albinism and immune deficiency [27]. Mutations in Rab39B, a Golgi-localized neuronal Rab that may play a role in synaptic maintenance, are responsible for X-linked mental retardation [28]. In addition, Rab38 [mutated in rat's Ruby (red eyed dilution; R) locus and the homologous mouse chocolate (cht) locus] has been implicated in the autosomal recessive disorder Hermansky–Pudlak syndrome (HPS) that is characterized by pigmentation and blood clotting disorders [29]. Recently, mutations in several HPS genes which encode components of Biogenesis of lysosome-related organelles complex-3 (BLOC-3), a Rab32 and Rab 38 GEF, have also been identified. It is postulated that the resulting defects in the biogenesis of lysosomal related organelles, of which Rab38 is believed to play a role, gives rise to some of the symptoms observed in HPS, including albinism and impaired platelet function [30]. Rab mutations or problems that are associated with aspects of Rab-mediated transport may indeed underlie a wider spectrum of neurological [31–33] and immune disorders [34].

As Rabs modulate membrane trafficking of growth factor receptors and cell adhesion molecules, it is also conceivable that dysregulation with regard to Rab-mediated endocytosis or recycling could lead to failure to control cell proliferation, adhesion and migration. A good number of Rabs have also been associated with human cancer [35,36]. Interestingly, cellular transformation and invasion are linked largely to changes in expression levels of these Rabs, rather than their mutations. Rab31, a member of the Rab5 subfamily, has recently emerged as a membrane traffic modulator that has interesting associations with breast carcinoma as well as glioma, and is the focus of this review. We first take a broad overview at our current understanding of Rabs that have been implicated in cancer (see summary in Table 1).

**Table 1:** A summary of studies implicating Rabs in cancer

Rab	Known physiological role	Implication in cancer	References
Rab1A	ER-Golgi transport, autophagy	Elevated in tongue squamous cell carcinomas and melanoma	[37,38]
Rab2	ER-Golgi transport	Elevated in peripheral blood mononuclear cells (PBMCs) of tumour bearing patients	[39,40]
Rab5A	Endocytosis	Elevated in non-small cell lung carcinoma, autonomous thyroid adenomas, hepatocellular carcinoma and ovarian cancer	[43–46]
Rab5B	Endocytosis	Elevated in melanoma cells	[38]
Rab5C	Endocytosis	A role in enhancing EGF-induced invasion by breast cancer cells	[47]
Rab7	Endo-lysosomal transport	Elevated in autonomous thyroid adenomas, associated with prostate cancer progression	[44,48]
Rab8	Polarized exocytosis	Regulates exocytosis of MT1-matrix metalloproteinase	[76]
Rab20	Endocytosis/phagocytosis	Elevated in pancreatic carcinoma	[49]
Rab23	Modulation of Sonic hedgehog signalling	Elevated in hepatocellular carcinoma and diffuse-type gastric cancer	[51,52]
Rab25	Endosomal recycling	Associated with various aspects of breast, ovarian, oesophageal, and bladder cancers, as well as head and neck squamous cell carcinoma	[59–63]
Rab27B	Regulated secretion/exocytosis	Marker for breast cancer progression, invasiveness and metastasis	[53–55]
Rab31	EGFR endosomal trafficking, M6PR trafficking from TGN to late endosome	Elevated in breast cancer and influences breast cancer, cervical cancer and glioblastoma progression	[93–99]
Rab32	Melanosome transport, mitochondrial dynamics, autophagy	Tumourigenesis of neuroendocrine tumours	[66]

## Rabs in human cancer

Dysregulated expressions of multiple Rabs spanning the entire exo-cytic and endocytic pathways have been shown in transcription profiling analysis of various cancer tissues [35,36]. Rab1A, which regulates ER-Golgi transport, is elevated in tongue squamous cell carcinomas [37] and melanoma [38]. Rab2, which also functions in the ER-Golgi boundary, is elevated in peripheral blood mononuclear cells (PBMCs) of tumour bearing patients [39,40]. Rab5 isoforms (Rab5A, -B and -C) are key regulators of the early endocytic pathway, and are known to profoundly influence cell motility and invasion, possibly through the regulation of β1-integrin traffic [41,42]. Rab5A has been shown to be up-regulated in non-small cell lung carcinoma [43], autonomous thyroid adenomas [44], hepatocellular carcinoma [45] and ovarian cancer [46]. Rab5B expression is elevated in melanoma cells [38]. Rab5C plays a role in enhancing EGF-induced invasion by breast cancer cells [47]. The key late endosomal Rab, Rab7, has been shown to be up-regulated in autonomous thyroid adenomas [44] and implicated in prostate cancer progression [48], possibly through its down-regulation of growth factor receptor signalling and its regulation of the movement of lysosomes, which carry proteinases that aid in cell motility. Rab20 is overexpressed in exocrine pancreatic carcinoma [49], and silencing of Rab20 reduced hypoxia-induced apoptosis [50]. Rab23 is overexpressed in a fraction of hepatocellular carcinoma [51] and diffuse-type gastric cancer [52]. Rab27B is involved in multiple aspects of breast cancer progression and is a prognosis marker. It may act through its regulation of the exocytosis of vesicles carrying Heat shock protein (HSP)-90α, which in turn activates matrix metalloproteases that aid in invasiveness [53–55].

Members of the Rab11 subfamily (Rab11A, Rab11B, and Rab25) are key regulators of endocytic recycling, including that of integrin, and their dysregulation are likely to affect aspects of cell transformation and migration [56]. Rab25 [57,58], a member of the Rab11 subfamily that is highly expressed in the epithelial cells of the gastrointestinal tract, lungs and kidney, has in the past few years been implicated in cancers from multiple organs. These include breast [59,60], ovarian [59], oesophageal [61], bladder [62] as well as head and neck squamous cell carcinoma [63]. The Rab coupling protein (RCP) or Rab11 family interacting protein 1 (Rab11FIP1), which is a Rab25 effector, is also well known as a breast cancer promoting gene [64]. Rab25's role in cancer is in some cases enigmatic as it could appear to act either as a cancer and metastasis promoter or a tumour suppressor, and we have previously suggested that its mode of action may depend on the availability of its effector RCP [65].

There are a number of other Rabs for which changed expression levels are associated with various types of cancers, although their mechanism of action has not yet been speculated upon. Rab32 dysregulation may be involved in tumourigenesis of neuroendocrine tumours [66]. Rab36 resides in a portion of chromosome 22q11, which is frequently deleted either heterozygously or homozygously in paediatric brain rhabdoid tumours [67].

Activities of Rabs in association with cancer have, in some cases, been shown to be epigenetically modulated. For example, down-regulation of Rab37 in metastatic lung cancer could be due to of promoter hypermethylation [68]. The micro-RNA (miR)-50 inhibits autophagy and tumour growth in colon cancer cells by suppressing, among other genes, the expression of Rab1B [69], the latter being an important factor in the initiation of autophagy [70]. Another miR, miR-451, has tumour suppressor functions in human non-small cell lung cancer, and could act by suppressing the expression of Rab14 [71]. On the other hand, miR-373 could be down-regulated by aberrant promoter methylation in colon cancer, with a concomitant up-regulation of its target, Rab22A [72]. miR-200b is a prognostic factor of breast cancer [73] and glioma [74], and it targets multiple Rabs including Rab3B, Rab18, Rab21 and Rab23.

Rab are not conventionally denoted as either oncogenes or tumour suppressors. However, abnormal expression of Rabs could conceivably drive several aspects of cellular transformation, particularly mitogenic signalling and cell migration/invasion. Multiple endocytic Rabs influence trafficking and signalling of cell surface growth factor receptors. Impaired or altered Rab regulation of the endocytic itineraries of these receptors could lead to impairment in receptor recycling or degradation, thus promoting mitogenic signalling that pre-disposes cells to oncogenic transformation [35,75]. On the other hand, Rab-mediated endocytosis and recycling is linked to cell migration and invasion. Rab8, for example, regulates exocytosis of MT1-matrix metalloproteinase (MT1-MMP), a key metastatic factor, to invasive structures [76]. Rab25 appears to promote invasive migration in a 3-dimensional matrix by associating with and mediating the recycling of α5β1 integrin and the epidermal growth factor receptor (EGFR), likely acting through RCP [77–79]. In the next section, we outline how Rab31 has been implicated in human cancers, and in the section after postulate the underlying mechanisms based on recent findings.

## Rab31 and findings implicating Rab31 in human cancers

Rab31 was first cloned from human melanocytes and named Rab22b based on its close homology with Rab22 [80], but was also named Rab31 when subsequently cloned from human platelets [81]. Rat (rRab22b) [82] and mouse orthologues [83] were also subsequently reported. Structurally, Rab31 is homologous to Rab5 and is grouped under the Rab5 subfamily [84,85]. Larocca and colleagues first showed that Rab31 transcripts are enriched in brain oligodendrocytes, and demonstrated by video microscopy that Rab31 prominently labels tubulovesicular carrier structures originating from the trans-Golgi [82], and that Rab31 regulates transport of the cation-dependent mannose 6-phosphate receptor (CD-M6PR) from the Golgi to the endosome [86]. The authors also showed that Rab31 interacts with the Lowe oculocerebrorenal syndrome protein OCRL-1 (an Inositol polyphosphate 5-phosphatase) and that this interaction is required for trans-Golgi network (TGN) organization and transport carrier formation [87].

We have developed Rab31 antibodies that supported an enrichment of Rab31 protein in brain tissues and a functional role for Rab31 at the TGN [83,88]. In addition, our findings also suggested that Rab31 has a role in regulating early endosome-late endosome transport, particularly of the epidermal growth factor receptor (EGFR) [88]. Other than M6PR and EGFR, Rab31 has also recently been shown to bind the signalling adaptor protein, phosphotyrosine interaction, pleckstrin homology (PH) domain, and leucine zipper-containing protein (APPL) 2 [89]. Two proteins GAPex-5 [90] and Rin3 [91], have been identified as GEFs for Rab31, and one of its confirmed effectors is early endosome antigen 1 (EEA1), which it shares with Rab5 and Rab22.

Several expression profiling analyses have implicated Rab31 in human cancers. A Serial Analysis of Gene Expression (SAGE) profiling found Rab31 to be among 11 genes that are robustly overexpressed in samples of Oestrogen Receptor α (ERα) positive breast carcinomas [92]. ERα is a transcription factor that is activated by oestrogens such as oestradiol, and regulates the transcription of target genes by binding to the oestrogen response element (ERE) upstream of the target genes. Rab31 transcripts were also found to be elevated in breast cancer cells expressing the urokinase-type tissue plasminogen activator (uPA)-receptor splice variant uPAR-del4/5 [93–95], and high Rab31 levels were significantly associated with distant metastasis-free survival and overall survival [96]. An analysis in advanced ovarian cancer samples did not, however, reveal any significant association with overall or progression-free survival [97]. Rab31 is also among the genes that associate with tumour progression in centrosomal protein transforming acidic coiled coil (TACC) 3 overexpressing HeLa cells as a model of cervical cancer [98].

Other than breast cancer, Rab31 is also identified as one of the cohort (race)-dependent associations with glioblastoma survival [99]. Meta-analysis of microarray studies using Bayesian network analysis also found Rab31 to be among 10 genes that are most influential in the development of glioblastoma multiforme [100].

## Mechanisms underlying Rab31's role in cancer

Several recent findings have helped to shed light on the possible underlying molecular pathways and mechanisms linking Rab31 to cancer. These are outlined and discussed below, headed by key questions pertaining to Rab31's expression and pathophysiological roles.

### Why are Rab31 levels elevated in cancer cells?

**Fig. 1:**
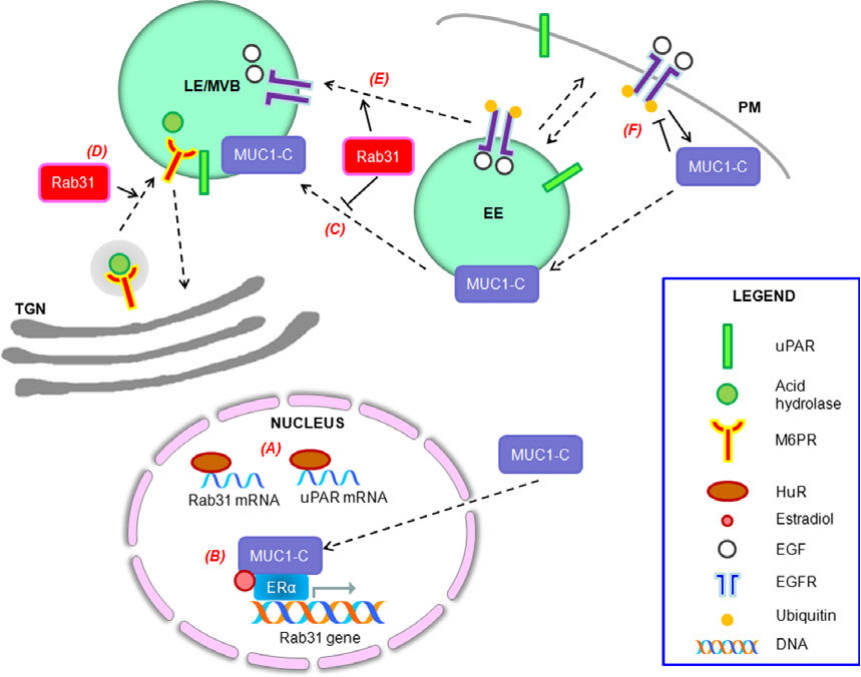
Rab31 and the interactions implicated in its role in cancer. (A) HuR stabilizes transcripts including that of Rab31 and uPAR. (B) Transcription of Rab31 is regulated by an ER responsive element. MUC1-C stabilizes and activates ERα, which in turn activates Rab31 transcription in an estradiol-dependent manner. (C) Rab31 inhibits the lysosomal degradation of MUC1-C, *via* an as-yet-undefined mechanism, thus elevating MUC1-C levels. (D) Rab31 regulates the movement of M6PR from the TGN to endosomes. M6PR, in turn, interacts with uPAR and may be responsible for its movement to endosomes for degradation. (E) Rab31 participates in the trafficking of ligand-bound EGFR from early to late endosomes, thus enhancing the rate of degradation of ligand-bound EGFR. (F) MUC1-C is phosphorylated by ligand-bound EGFR, leading to enhanced interaction with downstream signalling components. In turn, MUC1-C inhibits the ubiquitination of EGFR, thus potentiating its signalling. Dashed lines represent movement of proteins. Solid arrows represent activating mechanisms of action; solid blocked arrows represent inhibiting mechanisms of action. TGN, trans-Golgi network; EE, early endosome; LE/MVB, late endosome/multivesicular body; PM, Plasma membrane.

Rab31 levels are elevated in breast cancer cells, and recent findings offer two explanations for the phenomenon. One possibility is explained by a recent discovery that Rab31 transcripts are targets of HuR [101], an mRNA-binding and stabilizing protein of the ELAV-Hu family [102] which could thus stabilize Rab31 transcripts, resulting in their elevated levels (Fig. 1A). HuR itself is notably overexpressed in breast cancer tissues and has prognostic value [103,104]. Another possibility is related to the observation that Rab31 is selectively elevated in ERα-positive breast cancer samples [92]. A new key finding in this regard is that the Rab31 promoter region has an ER responsive element [105], and could be thus regulated or deregulated in breast cancer cells by trans factors associating with the element. One such factor turned out to be mucin-1 (MUC1), an oncogenic glycoprotein that has been shown to be expressed in a large fraction of breast cancer samples [106].

MUC1 is a heterodimeric transmembrane protein consisting of MUC1-N (which harbours mucin-like repeats) and MUC1-C, which spans the cell membrane [107]. MUC1-C could be internalized by clathrin-based endocytosis [108] and imported into the nucleus [109], where it stabilizes and activates ERα [110]. Jin and colleagues demonstrated that MUC1-C forms a complex with ERα, and could activate Rab31 transcription in an oestradiol-dependent manner (Fig. 1B). Up-regulated Rab31 could in turn elevate MUC1-C levels, probably by reducing its lysosomal degradation through an as-yet-undefined mechanism (Fig. 1C). Rab31 expression in MCF10A cells could promote the formation of anchorage-independent cytospheric structures (mammospheres) in a MUC1-C-dependent manner [105]. MUC1-C and Rab31 therefore appear to form an auto-inductive loop that results in sustained over-expression of MUC1-C in breast cancer cells. Such an auto-inductive regulatory loop has also been previously reported between MUC1-C and the Signal transducer and activator of transcription 3 (STAT3), which is also a promoter of malignancy [111].

### Is Rab31 a driver in cancer, and if so, how?

Interestingly, when Rab31 is overexpressed in breast cancer cell lines, it enhanced proliferation and diminished cell adhesion towards several extracellular matrix (ECM) components, as well as attenuated cell invasion through Matrigel [112]. When breast cancer cells moderately overexpressing Rab31 were xenografted onto nude mice, these exhibited significantly reduced lung metastasis compared to control cells. In invasive tumour lines at least, high levels of Rab31 appear therefore to switch these cells from an invasive to a more proliferative phenotype [112].

Could any of the factors that help elevate Rab31 levels described above attest to Rab31's oncogenic potential? Other than Rab31, HuR also stabilizes the transcript of uPA and its receptor (uPAR) [113]. uPA, localized by uPAR to the plasma membrane, cleaves plasminogen to give plasmin, which in turn cleaves and activates matrix metalloproteases (MMPs) that aid in degradation of ECM components. uPAR is elevated during inflammation and ECM remodelling, and is usually associated with poor cancer prognosis [114]. uPAR could also activate a variety of intracellular signalling pathways such as the mitogen activated protein kinase (MAPK) pathway and the phosphatidylinositol 3-kinase (PI3K)-Akt pathway [115] through the engagement of co-receptors, such as integrins [116]. A splice variant of uPAR, uPAR-del4/5, which is unable to bind uPA, is a known prognostic marker for breast cancer [94,96,113] although phenotypically, *in vitro*, cells overexpressing this variant appear to have reduced invasive properties [95,117], much like that observed for Rab31 [112]. While Rab31 transcripts have also been found in cancer cells with the uPAR splice variant, and both are stabilized by HuR, it is unclear if the two have a causal or functional relationship. An interesting connection with Rab31 in this regard is that uPAR interacts with CI-M6PR [118,119], whose TGN-endosome transport is regulated by Rab31 [86,87]. The interaction of uPAR with CI-M6PR may regulate the movement of uPAR to endosomes for degradation and thus modulate the levels of uPAR on the cell surface [118] (Fig. 1D). How this uPAR-CI-M6PR connection might relate to Rab31's role in cancer is yet unclear. It is, however, conceivable that Rab31 may modulate uPAR's activity in signalling as well as ECM modulation, *via* its regulation of M6PR trafficking dynamics, thus impacting on tumourigenesis and invasion.

Notably, the other modulator of Rab31 expression, MUC1-C, is a multifunctional oncoprotein [107]. MUC1-C overexpression in fibroblast is sufficient to induce anchorage-independent growth and tumour formation in nude mice [120]. In the nucleus, MUC1-C activates the Wnt/β-catenin [121], STAT3 [111] and NF-κB [122]- based transcription, all of which have been associated with tumourigenesis. At the plasma membrane, MUC1-C interacts with EGFR [123–125] and perhaps other members of the ErbB family to activate the MAPK and PI3K-Akt pathways. Furthermore, MUC1 could promote autophagy and survival responses of cancer cells to nutrient deprivation [126]. It was also recently shown to stabilize and activate hypoxia-inducible factor-1α (HIF-1α) to regulate hypoxic response in pancreatic cancer cells [127]. In view of the oncogenic potency of MUC1, it does appear that if Rab31 could effectively elevate and sustain functional levels of MUC1-C, it could help drive oncogenesis.

### Rab31's modulation of EGFR trafficking – could it be tumour suppressive?

A twist to the general plot above came about from our findings that Rab31 directly modulates EGFR trafficking and possibly its signalling [88,128]. A hint that Rab31 may regulate EGFR trafficking first came from two earlier reports, the first from Stahl's laboratory which showed that GAPex-5, then newly identified as a Rab5 GEF, modulates EGFR ubiquitination, trafficking and degradation [129]. Another report indicated that GAPex-5 is also a GEF for Rab31, and it regulates insulin-stimulated Glut4 translocation to the plasma membrane in adipocytes [90]. We found that silencing of Rab31 inhibited, while overexpression enhanced, EGFR trafficking to the late endosomes (Fig. 1E), and the former observation phenocopied the effect of GAPex-5 silencing on EGFR trafficking. Interestingly, Rab31 is associated with EGFR in a GTP-dependent manner, and this association is dependent upon its effector early endosome antigen 1 (EEA1) as well as GAPex-5 (but not Rin3 [91], another Rab31 GEF) [128]. Rab31 may thus be recruited as part of an EGFR-containing membrane trafficking complex to regulate its transit from the early to late endosomes. Overexpression of Rab31 appeared to enhance the degradation of EGFR, and we have observed that, for A431 cells at least, this translates to a moderate decrease in the rate of cell proliferation [88].

On the face of it, our observations in A431 cells run counter to what might be expected for Rab31 overexpression in breast cancer cells. Given that Rab31 overexpression in breast cancer cell lines increased their proliferation [112], the discrepancy may be down to cell type differences. It may also simply be just another illustration of the complexity associated with cancer cells and tissues, where multiple factors act along with, or counter the action of one another in intersecting pathways. The cancerous phenotype, and its different dynamic manifestations as the cancer progresses, is thus a combinatorial sum of many. It is difficult at the moment to gauge quantitatively which one of the two apparently contrasting actions of Rab31, namely the auto-inductive loop that it is engaged with MUC1-C, or its effect on EGFR trafficking and signalling, would be a more important determinant of the cancer phenotype. It is conceivable though that one important determinant in the complex equation would be the availability and activity of its GEF GAPex-5, as well as it is yet to be identified GAP(s). In a way reminiscent to Rab25, both the availability of regulators and effector would be important variables in determining if the Rab would be oncogenic, or conversely tumour suppressive [65]. Furthermore, in breast cancer in particular, any moderating effect of Rab31 on EGFR signalling may well be completely muted by the fact that EGFR family receptor tyrosine kinase and their mutants are prevalent [130], or the activation of competing recycling pathways that will recycle endocytosed EGFR back to the surface [78].

On the other hand, one should also keep in view the complex relationship between Rab31, EGFR and MUC1-C. MUC1-C itself interacts with EGFR and is indeed a substrate of EGFR tyrosine kinase activity [123,124], with the phosphorylation of MUC1-C by EGFR leading to enhanced interactions with its downstream components [123]. Conversely, MUC1-C was shown to inhibit ubiquitination of ligand-bound EGFR, thus reducing the degradation and enhancing the recycling of EGFR to the cell surface, thus potentiating its signalling [124] (Fig. 1F). At the moment it is unclear how elevated Rab31 levels may affect this EGFR-MUC1 interaction, but it is conceivable that the presence of Rab31 could alter EGFR signalling in this regard. Further work should determine if this influence is positive or negative. Given that overexpression of Rab31 in breast cancer cell lines actually enhanced proliferation [112], Rab31, when elevated in the presence of MUC1-C may enhance instead of retard EGFR signalling in these cells. Another point of contention that requires further clarification pertains to the effect of Rab31 on MUC-1C's expression. Jin and colleagues surmised that Rab31 could have diminished MUC-1C's lysosomal degradation as the lysosome inhibitor chloroquine increased MUC1-C levels in Rab31 silenced cells. How then does Rab31 diminish late endososome-lysosome targeting of MUC1-C while increasing the transport of EGFR? Answers to these and other questions await resolution by further work.

## Epilogue and future perspectives

In this brief review, we have discussed how recent findings may explain Rab31's elevation in cancer, and how elevated Rab31 may influence cancer cell signalling through its effect on EGFR endosomal traffic. To fully understand the significance of Rab31 as a prognostic marker, we posit that assessment of the levels of its regulators such as GAPex-5 in various cancers would be important. It is far too early to postulate if Rab31 and its regulators would have therapeutic values. However, it is conceivable that in breast cancer cells overexpressing EGFR or other ErbB family members, using Rab31 to attenuate EGFR signalling may be a potentially useful adjunct therapy to anti-EGFR drugs. This may attenuate the selection pressure that would lead to the development of resistance against drugs targeting EGFR [131,132].
